# Chronic Kidney Disease stratification using office visit records: Handling data imbalance via hierarchical meta-classification

**DOI:** 10.1186/s12911-018-0675-x

**Published:** 2018-12-12

**Authors:** Moumita Bhattacharya, Claudine Jurkovitz, Hagit Shatkay

**Affiliations:** 1 Computational Biomedicine Lab, Computer and Information Sciences, University of Delaware, Newark, DE USA; 2 Value Institute, Christiana Care Health System, Newark, DE USA; 3 Center for Bioinformatics and Computational Biology, Delaware Biotechnology Inst, University of Delaware, Newark, DE USA

**Keywords:** Imbalanced data, Meta-classification, Hierarchical classification, Electronic health records, Kidney disease

## Abstract

**Background:**

*Chronic Kidney Disease* (*CKD*) is one of several conditions that affect a growing percentage of the US population; the disease is accompanied by multiple co-morbidities, and is hard to diagnose in-and-of itself. In its advanced forms it carries severe outcomes and can lead to death. It is thus important to detect the disease as early as possible, which can help devise effective intervention and treatment plan.

Here we investigate ways to utilize information available in electronic health records (EHRs) from regular office visits of more than *13,000* patients, in order to distinguish among several stages of the disease. While clinical data stored in EHRs provide valuable information for risk-stratification, one of the major challenges in using them arises from *data imbalance*. That is, records associated with a more severe condition are typically under-represented compared to those associated with a milder manifestation of the disease. To address imbalance, we propose and develop a sampling-based ensemble approach, *hierarchical meta-classification*, aiming to stratify CKD patients into severity stages, using simple quantitative non-text features gathered from standard office visit records.

**Methods:**

The proposed hierarchical meta-classification method frames the multiclass classification task as a hierarchy of two subtasks. The first is binary classification, separating records associated with the majority class from those associated with all minority classes combined, using meta-classification. The second subtask separates the records assigned to the combined minority classes into the individual constituent classes.

**Results:**

The proposed method identifies a significant proportion of patients suffering from the more advanced stages of the condition, while also correctly identifying most of the less severe cases, maintaining high *sensitivity*, *specificity* and *F-measure* (*≥ 93%*). Our results show that the high level of performance attained by our method is preserved even when the size of the training set is significantly reduced, demonstrating the stability and generalizability of our approach.

**Conclusion:**

We present a new approach to perform classification while addressing data imbalance, which is inherent in the biomedical domain. Our model effectively identifies severity stages of CKD patients, using information readily available in office visit records within the realistic context of high data imbalance.

## Background

*Chronic kidney disease* (*CKD*) is defined as kidney damage persisting for more than three months. It is currently affecting about *15%* of the adult population in the US, accompanied by co-morbidities and associated with increased mortality rates [1]. The disease is typically classified into five stages, *1*–*5*, indicating increasing order of severity [2]. These severity stages are clinically quantified through the use of the *Estimated glomerular filtration rate* (*eGFR*), an indicator of the level of kidney function.^1^ The glomerular filtration rate is estimated from *serum creatinine lab tests*, *race*, *sex* and *age*. As chronic kidney disease – even in its advanced stages – is often *asymptomatic*, the relevant lab tests are not typically ordered and many CKD patients go undiagnosed [3]. Patients who remain under-treated, especially in stages *4* and *5*, are at high risk for end-stage renal disease and death.

A study reported by the Kidney Early Evaluation Program (KEEP) [4], indicates that fewer than 30% of the *122,502* patients enrolled in the program at stages *4* and *5* have ever been seen by a nephrologist. Notably, *95*% of the enrolled patients did visit their general practitioner during the year preceding the study, for other conditions. As such, developing a risk stratification model based on information gathered during these office visits, which can separate CKD patients into severity stages, can be used to alert general practitioners about a patient’s risk for advanced stage (*4* or *5*) CKD, prompting the physician to order the lab tests needed to confirm the diagnosis.

A number of recent studies have employed machine learning methods to stratify patient risk and predict the onset of a disease [5–9]. These studies have typically used lab test results, insurance information and narrative text, along with office visit records, whereas our study solely utilizes simple attributes that are routinely collected and can be found in readily available office visit records. Additionally, the datasets used in most of these studies are an order of magnitude smaller *(< 2,300* records) [5–7] compared to the one used in our study. These studies also do not impose the inclusion of only temporally early records in the training sets, and testing on later records, as we do in our work here. Notably, none of these studies handle class imbalance that is inherent in the dataset, as we do here. Ours is the first study that aims to identify disease severity levels solely using simple quantitative non-text attributes collected during patient’s office visit, while directly addressing imbalance in the number of records available across severity stages. Utilizing standard office visit records allows our approach to be broadly applicable to most patients who routinely visits a physician.

Collaborating with physicians from Christiana Care Health System, the largest health-system in Delaware, we analyze a dataset gathered from *13,111* patients who had been seen in primary care or specialty practices over a nine-year period. Data from Nephrology Practices EHR are not included in this dataset. The dataset comprises information collected during patients’ visits to multiple primary care and specialty practices across Delaware. The individual records each consist of *495* simple quantitative non-text attributes summarizing a patient’s *demographics, vital signs*, *diagnosed conditions* and *medications*. We represent each patient’s visit record using the values of these attributes as features. In contrast to text-based physician notes that are not always available or comprehensive, these *495* non-text attributes are available for the vast majority of patient. Moreover, unlike natural-language physician notes – whose analysis is the topic of much current research in medical informatics – the semantics of the *495* non-textual attributes is unambiguous and readily interpretable. The dataset is further described in the Methods section.

While clinical data stored in EHRs provide valuable information for patient risk stratification, one of the major challenges in using them arises from *data imbalance*. Explicitly, manifestation of the most severe conditions is relatively rare in the patient population, while a majority of patients either exhibit mild manifestation or may not even show any signs of the condition. In particular, our dataset includes *10* times more records associated with *stage 3* than records associated with *stage 4*; the proportion of *stage 3* to *stage 5* records is even larger, namely, *23:1.* Imbalanced datasets, characterized by a skewed class distribution, are common in quite a few challenging data mining applications, ranging from gene-finding, through epidemiology to fraud-detection, where the class of interest is severely underrepresented in the population with respect to the other classes. Classifiers learned from such an imbalanced dataset using off-the-shelf packages typically show poor performance in identifying minority class records (in our case – the class usually associated with the more severe condition), as demonstrated in the Results section. Thus, addressing imbalance is critical for correctly identifying the important records associated with the minority class.

We thus propose and develop a *meta-learning* based *hierarchical classification* approach that addresses data imbalance, while performing multiclass classification. We utilize the proposed method to stratify a set of CKD patients already identified as *stage 3* or higher, into severity stages (*3*–*5*), using information gathered from standard office visit records. Our method effectively identifies a significant proportion of patients suffering from the more severe conditions (stages *4* and *5*), attaining high *sensitivity*, while also correctly identifying most of the less severe cases (*stage 3*), maintaining a high level of *specificity*.

The machine learning literature proposes to handle data imbalance through either under-sampling or over-sampling strategies [10–16]. The former involves reducing the majority class by removal of instances from the training set, while the latter over-samples with repetition from the minority class – thus increasing its impact within the training process. Several variations of under- or over-sampling were proposed in previous studies, including one-sided selection [10] and *synthetic minority oversampling technique* (*SMOTE*) [11]. It is important to note that most of these studies were done in the context of binary classification, while the task we address here is a multiclass task, where we aim to label each record with one of *three* possible stages.

Multiclass classification is usually addressed through conversion into multiple binary-classification tasks, employing either a *one-against-all* (*OAA*) or a *one-against-one* (*OAO*) approach [12]. Tan et al. [13] employ both to identify types of protein folds, while using rule-based learners to improve coverage of the minority class. Also in the context of protein classification, Zhao et al. [14] use OAA, while addressing data imbalance by employing under-sampling and SMOTE techniques. In an earlier work [15], cost sensitive ensemble methods have also been employed toward addressing class imbalance.

Before proposing our own method, we have utilized versions of the above methods, specifically, random under-sampling and over-sampling using *SMOTE*, within the OAA scheme (See Results section). None has improved on the results obtained by simple classifiers that do not account for class imbalance (to which we refer as *baseline classifiers*), such as simple random forest. Thus, as mentioned earlier, we develop and present a multiclass classification method, *hierarchical meta-classification*, aiming to stratify CKD patients into severity levels (stages *3–5*), while addressing data imbalance. Unlike approaches that utilize under-sampling and ignore much of the majority data, or approaches that use over-sampling, which create a large amount of mock-up data that can lead to over-fitting, our approach neither ignores data nor creates synthetic samples, yielding higher level of performance, while avoiding over-fitting.

We frame the multiclass classification task as a hierarchy of two subtasks. We first aim to separate the majority class records, namely those associated with stage *3*, from the combined class consisting of records associated with stages *4* and *5* (the minority classes). This binary classification task is addressed via meta-classification, which combines results obtained from an ensemble of multiple simple classifiers (*base-classifiers*) into a single classification decision [17]. In the second sub-task, we aim to separate the records labeled under the combined *stages 4 and 5* class, and assign each of these into its correct respective stage-based class, namely, either *stage 4* or *stage 5*. Ours is the first study that utilizes meta-classification in combination with a hierarchical approach to address data imbalance.

Training of the hierarchical meta-classifier utilizes the set comprising the earlier office visit records, collected throughout *2007–2014*, while the test set is kept fixed to include only records collected in *2015*. We use multiple evaluation measures to assess the performance of our method, namely, *overall accuracy*, *specificity, sensitivity* (aka *true positive rate*)*, precision* and *F-measure* [18]. Our results show that the proposed method trained on a dataset represented via the complete set of features, performs at a level at or above *93%*, with respect to all evaluation metrics, and improves upon the baselines. The high-level of performance of our model demonstrates that patient information that are routinely collected during office visits form a sound basis for CKD risk stratification.

To assess the robustness and stability of our proposed approach in the face of data reduction, we gradually decreased the number of records included in the training set by pruning early years of patient history, one year at a time, giving rise to eight distinct training sets. We used the records collected in *2015* as the test set for assessing the performance of our model trained on each of the eight training sets, ensuring that the training set always contains temporally earlier records than the test set. Our results show that the classifier retains its good performance even when applied to datasets of varying sizes containing records gathered over a limited range of years, demonstrating the stability and generalizability of our proposed strategy.

## Methods

### Dataset

We used data collected from *13,111* patients across Delaware, during visits to primary care and specialty practices. The dataset consists of *120,739* records comprising patient information stored in the EHR; records were included in the dataset if the corresponding patient was diagnosed with *CKD* stage *3* or higher during any follow-up visit, (as indicated by a eGFR value  < *60 mL/min/1.73m*^2^). The dataset thus comprises all records of patients at stages *3*,*4* and *5*.^2^ Of the *120,739* records, *27,521* records missed values for one or more pertinent attributes, and were thus removed from the dataset; the remaining set of *93,218* complete records is used in this study.

The key characteristics of this record set are summarized in Table 1, while Table 2 summarizes the three categories of features included in the records while showing the number of features for each category. Values for all these features are readily available, as they are regularly recorded during routine office visits and stored in the EHR. As such, our approach generalizes well beyond CKD, and can be applied to cohorts of patients for whom information is recorded in the context of other disease. We note that in contrast to the earlier version of this work [19], we do not include here the patients’ *medications* as part of the feature set, thus reducing the number of features from the original *495* to *462*. Several medications prescribed to stage *3* patients can be harmful to patients at advanced stages of the condition (stages *4–5*). As such, medications can be indicative of a diagnosed disease stage, rather than predictive of it. To ensure our model is truly predictive, we have removed *medications* from the feature set. The latter actually leads to improved average specificity, and only slightly reduced sensitivity with respect to the advanced CKD stages.

**Table 1:** List of data characteristics along with their respective values in our dataset

Characteristic	Value
Number of Patients	13,111
Age Range (25^*th*^ –75^*th*^ Percentile)	60–80
Mean Age (σ)	70 (*12*)
% Female	60%
% Male	40%
Avg. Number of Visits per Patient	17

**Table 2:** The three variable-categories comprising our dataset. The categories themselves are listed on the left. The middle column shows the number of variables per category, while the right column provides a few examples of features included within the respective categories

Category	Number of features	Examples
Demographics	4	Gender; Age; Ethnicity; Race
Vital Signs	4	Heart Rate; Systolic and Diastolic Blood Pressure; Body Mass Index
Diagnosed Conditions	447	Benign essential hypertension; Type 2 diabetes mellitus; Obesity

We further reduce the feature set by excluding seven features that directly indicate CKD, resulting in a total of *455* features used for representing each patient’s record. These excluded features correspond to the following seven diagnosed conditions: *CKD stage 2, CKD stage 3, CKD stage 4, End Stage Renal Disease, Chronic Renal Failure, History of renal transplant (situation)* and *Renal Failure Syndrome*. Consequently, each record, r^k^ (*1* ≤ k ≤ *93,218*), is represented as a *455*-dimensional vector, ${V}^k=<{v}_1^k,\dots, {v}_{455}^k>,$ where each dimension corresponds to one of the *455* features.

As pointed out earlier, and as is typical within the biomedical context, the dataset used here is highly imbalanced, that is, the outcome we care most to identify (stages *4* and *5*) is rare and thus underrepresented. Specifically, within our dataset the ratio among the number of records associated with each of the stages *3*, *4* and *5* is *23*:*2*:*1*, respectively.

In our experiments, we examine the impact of using fewer and more recent records for stage prediction – as opposed to the complete patient history. As such, we experiment with eight progressively smaller datasets, in which each patient’s history within the year range *2007*–*2014* is truncated by removing from it one year at a time. Table 3 summarizes the eight resulting datasets, showing the distribution of records per class in each. The table also lists the number of records per stage as gathered in *2015*, where the latter is used as the test set throughout this study.

**Table 3:** Distribution of records among the three CKD stages within the datasets used in our study. The number of records associated with each of the three stages is shown for each of the eight training sets as well as for the test set. Each of the training sets listed was obtained by considering the records left in the dataset while progressively truncating the early years of patient history included (the range of years covered by each set is shown in the respective column header). The rightmost column provides the number of records per stage in the *test set*, which was fixed to contain records gathered during *2015*

CKD stages	Training set distribution								Test set
	2007–2014	2008–2014	2009–2014	2010–2014	2011–2014	2012–2014	2013–2014	2014	2015
Stage 3	73,425	72,808	70,127	65,326	57,863	46,881	33,072	17,273	8,419
Stage 4	6,976	6,903	6,579	6,060	5,385	4,439	3,101	1,624	782
Stage 5	3,241	3,184	3,052	2,821	2,515	2,068	1,471	767	375
Total	83,642	82,895	79,758	74,207	65,763	53,388	37,644	19,664	9,576

### Classification and handling of imbalanced data

We next outline the approach we develop for multiclass classification under data imbalance, while briefly describing the simple baseline classifiers used for comparison and the performance evaluation measures employed throughout our study.

#### Baseline classifiers

We use several common classification methods that do not directly handle data imbalance as a simple baseline for comparison. These classifiers include *naïve Bayes, logistic regression, decision tree,* and *random forests,* utilizing the one-against-all strategy. We refer to these classifiers as the *baseline*. They are all trained and tested on the same set of records describe earlier, while using the same *455* features to represent each record. We employ the *Python scikit-learn* implementation to train the baseline classifiers [20].

#### Hierarchical meta-classifiers (our proposed method)

We have developed a hierarchical meta-classification approach for assigning a CKD stage (in the range *3–5*) to a patient record in the face of high data imbalance. The motivation for using a hierarchical approach stems from the clinical characteristics of kidney disease stages. Specifically, patients diagnosed with either stage *4* or stage *5* (eGFR < *30*) demonstrate a critically reduced kidney function, while those at stage *3* (eGFR range of *30–60*) show only a moderate decline in kidney function [2]. As such, the hierarchical strategy first separates records associated with the combined class that consists of stages *4* and *5* cases (i.e. the more severe cases) from those associated with stage *3*. In a second step it then further separates the combined class into two individual subclasses.

For conducting the first step (to which we refer as *coarse classification*) under data imbalance, we employ *meta-classification* – a technique that enables coalescing class labels obtained from multiple different classifiers into a single unifying classification result. The second step (to which we refer as *refinement classification*) is attained by training a simple classifier to separate the combined class into individual stage *4* and stage *5* classes.

**Fig. 1:**
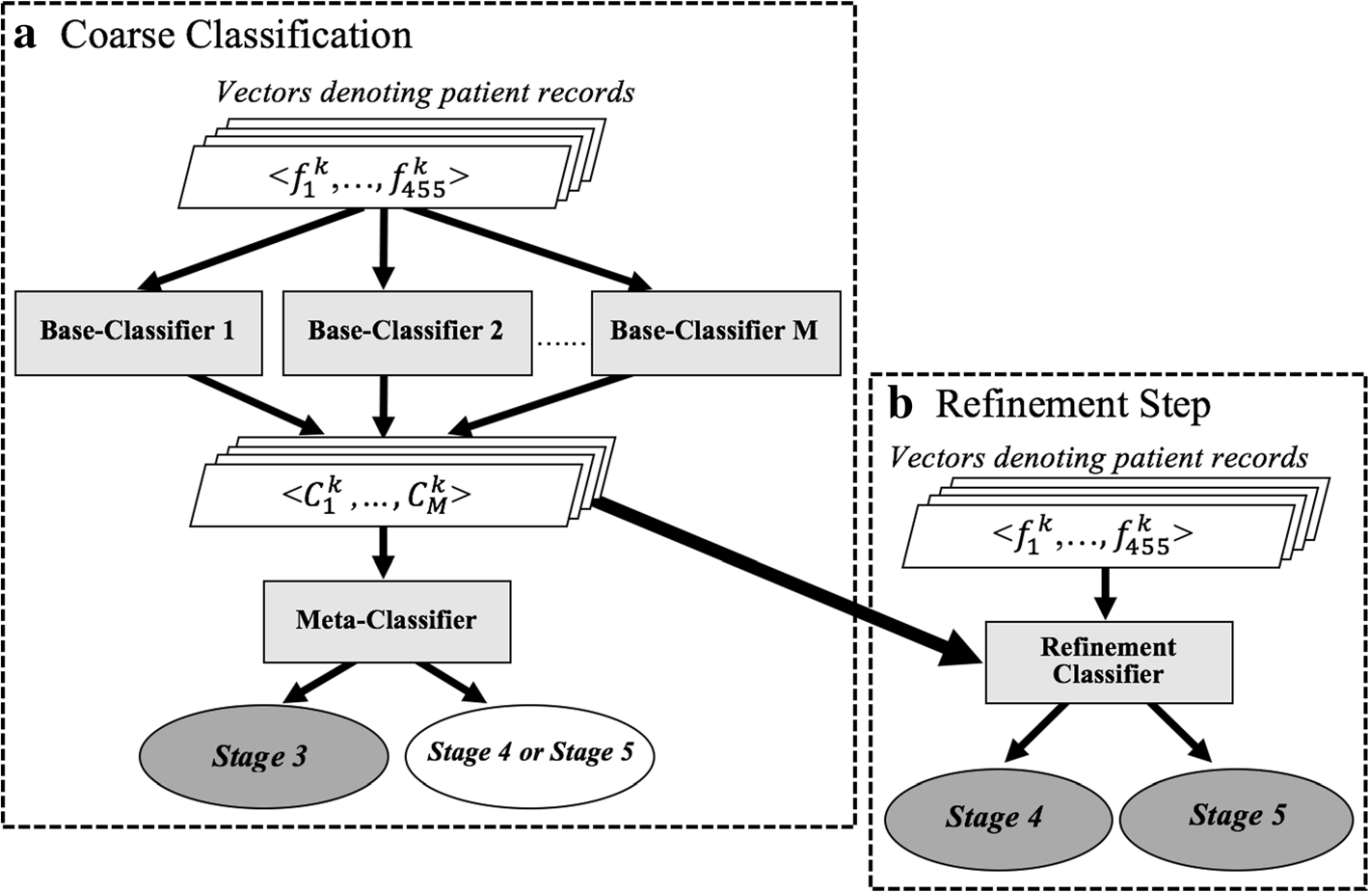
*Hierarchical meta-classification.* An overview of our sampling-based ensemble approach for multiclass classification while addressing data imbalance. **a** *Coarse classification*: The top dashed rectangle corresponds to the meta-classification scheme used to separate records associated with the combined class consisting of stages *4* and *5* from those associated with stage *3*. **b** *Refinement step*: The bottom dashed-rectangle corresponds to the classification step aiming to separate the combined class of advanced CKD stages (stages *4*&*5*) into its two individual constituent classes (stage *4* vs. stage *5*). In both steps, shaded rectangles represent the actual classifiers employed, the grey ovals denote the final classification outcome. The white oval in the Coarse Classification diagram represents the intermediate set of records, assigned to the combined class (stages *4&5*) by the meta-classifier, which is further split in the refinement step. The input to the base-classifiers and to the refinement classifier consists of *455-dimensional* vectors, representing the respective sets of patient records; the input to the meta-classifier comprises *M* dimensional vectors whose components correspond to the labels assigned by each of the *M* base-classifiers to the original (455-dimensional) vectors

Figure 1 illustrates this two-step approach. In the figure, the dashed-rectangle on the top/left depicts the coarse classification process of training/testing the multiple base-classifiers and the meta-classifier, while the bottom dashed-rectangle summarizes the process of training the refinement classifier which aims to separate the combined stage *4*&*5* class into its two individual constituent classes. The individual steps are further described below.

##### Coarse classification:

This step is key to addressing class imbalance and consists of two sub-tasks: 1) Training multiple simple classifiers (to which we refer as *base-classifiers*) on balanced datasets; and 2) Assembling the classification outcomes obtained from the base-classifiers into a single final classification result.

For the first sub-task, a set of *M* base-classifiers, {*C*_*1*_*,…,C*_*M*_ } are trained, and applied to each visit record, *r*^k^, where the latter is represented as a *455*-dimensional vector, <${v}_1^k$,…$, {v}_{455}^k$> as described earlier. Each base-classifier *C*_*j*_ (where *1 ≤ j ≤ M*) assigns a label ${C}_j^k$($\mathrm{where}\ {C}_j^k$is either *3* or *4–5*) to the vector *r*^k^. The *M* base-classifiers are trained on *M* balanced training sets. In our experiments *M* is set to *7* as explained later in this section. To produce a balanced set, we first sample at-random without replacement from the majority class (stage *3*, in our case) and then combine the sampled set with the complete set of minority class (the combined stages *4* and *5*, in our case). The number of records sampled from the data associated with the majority class is set to be the same as the total number of records within the minority class. We repeat the sampling and combining process *M* times to obtain *M* balanced training sets.

For the second sub-task, the class labels assigned by these *M* simple classifiers are used to re-represent the visit record *r*^*k*^ as an *M*-dimensional vector <${C}_1^k$*,…*${C}_M^k$>. This representation is then employed for training a meta-classifier that assigns a class label, *stage 3* or *stage 4–5,* to each record [17]. The meta-classifier thus treats the judgment from each base-classifier for each class as a feature value and uses these features for arriving at a final decision.

**Fig. 2:**
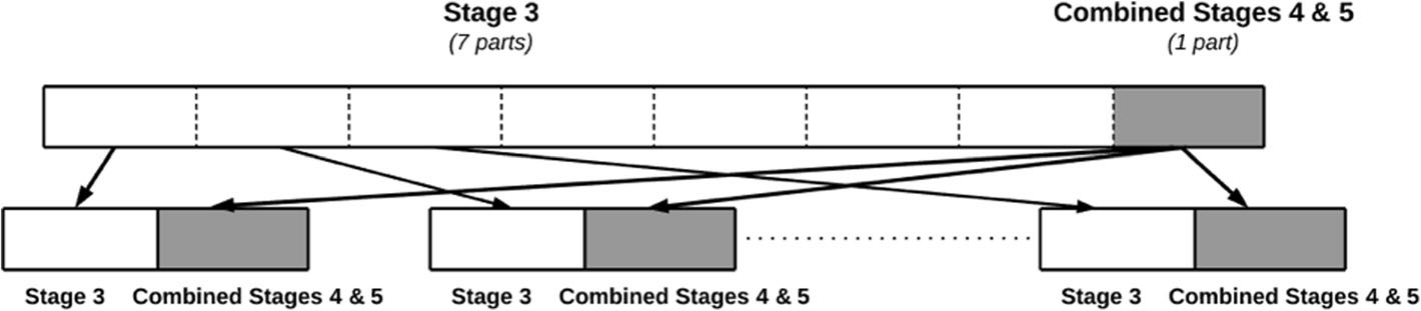
The partitioning scheme for obtaining balanced training sets, as a part of the hierarchical meta-classification approach. The record set associated with stage *3* is sampled at-random without replacement from the majority class to obtain *7* subsets (shown as white rectangles in the figure). Each subset contains the same number of records as that included in the set combining stages *4* and *5* (grey rectangles). Each of the sampled stage *3* subsets is paired with the set combining stages *4* and *5*, thus forming *7* balanced datasets in total, each having a balanced sample of stage *3* and stages *4*&*5* records

Specifically, in our dataset, as there are *7* times more records associated with stage *3* than with the combined set of stages *4* and *5*, we apply the sampling approach described above to the training set of stage *3* records, thus obtaining *7* stage *3* subsets (i.e. *M = 7*). We then combine each of these stage *3* subsets with the stage *4–5* set, forming a total of *7* datasets, each having a uniform distribution across CKD stage *3* and the combined stages *4–5* instances. Figure 2 illustrates the data partitioning scheme.

We train one base-classifiers on each of the resulting seven balanced training sets, giving rise to seven classifiers. We experiment with four commonly used simple classification methods, namely logistic regression, naïve Bayes, decision tree and random forest, to empirically choose the most suitable type of base-classifier. We have accordingly conducted four sets of experiments, each employing one of these four basic classification methods. We train each of the four classifier types on the *7* balanced sets, thus generating *7* base-classifiers per method. Using each set of *7* base-classifiers, we train a meta-classifier in which the training set was re-represented as *7-*dimensional vectors, where the value along the *i*^*th*^ dimension consists of the label obtained from the *i*^*th*^ base-classifier when applied to the original record representation. In all four sets of experiments the meta-classifier used is naïve Bayes, as it proved to perform best and has proven effective by others as well [21]. The resulting classifier aims to separate stage *3* records from records that are either stage *4* or stage *5* (see Fig. 1a).

##### Refinement classification step:

In this step, we separate the combined minority class records obtained from the coarse classification step (combined stages *4* and *5*, in our study) into the individual classes (as shown in Fig. 1b). To do so, we experiment with multiple simple classifiers, including random forest and naïve Bayes and show that the random forest classifier is most effective in separating records associated with stage *4* from those associated with stage *5*; as such this is the one we employ. We train the random forest classifier used in the refinement step, over the set of training records associated with stages *4* and *5*, under their original representation, as a *455*-dimensional feature vector per record.

We demonstrate in the Results section that our proposed scheme indeed significantly improves upon the baseline classifiers, the simple non-hierarchical meta-classifier as well as on previously reported methods to address imbalance, as all of the latter do not identify a significant proportion of stage *4* and stage *5* cases.

#### Testing and evaluation

To test the hierarchical meta-classifier, each of the base-classifiers is first applied to assign a class label to each record in the test set. The obtained labels are then used to form a feature vector, which becomes the input to the meta-classifier. The latter is applied to each newly represented vector thus separating stage *3* records from records of stages *4* or *5* (coarse classification step). Records classified into the combined stage-*4* and -*5* class are further categorized by the simple random forest classifier (refinement step), and assigned to either of the two individual classes, stage *4* or stage *5*.

To quantitatively assess the performance of all the classifiers with respect to each stage *i* (where *i* is *3, 4,* or *5* or the combined stages *4–5)*, we use the four common evaluation metrics, namely, *specificity*, *sensitivity* (also known as *True Positive Rate* or *Recall*), *precision* and *F-measure,* as defined below:

$$ \boldsymbol{Specificity}=\frac{TN_i}{TN_i+{FP}_i},\kern0.5em \boldsymbol{Sensitivity}=\kern0.5em \frac{TP_i}{TP_i+{FN}_i},\kern0.5em \boldsymbol{Precision}=\frac{TP_i}{TP_i+{FP}_i},\kern0.5em \boldsymbol{F}\hbox{-} \boldsymbol{measure}=2\cdot \frac{Precision\cdot Sensitivity\ }{Precision+ Sensitivity}, $$

where *TP*_*i*_ (*true positives*) denotes records of *stage i* that are correctly assigned to *stage i* by the classifier; *TN*_*i*_ (*True Negatives*) denotes records that are not associated with *stage i* and are not assigned to *stage i* by the classifier; *FP*_*i*_ (*False Positives*) denotes records not associated with *stage i* that are misclassified as *stage i*; while *FN*_*i*_ (*false negatives*) denotes *stage i* records that were incorrectly assigned to other stages by the classifier.

The next section provides a description of our experiments and results, demonstrating the effectiveness of our methods.

## Results

As described in the Methods section, in our experiments we employed as a baseline four simple classification methods that do not account for data imbalance, namely, naïve Bayes, logistic regression, decision tree and random forests. To handle the data imbalance, we first experimented with previously reported methods, namely, random under-sampling and over-sampling using SMOTE. We also applied simple meta-classification to the records to address imbalance (detailed description of the simple meta-classifier was discussed in the earlier, conference-version, of this work [19]). Since these methods failed to identify a large number of stages *4* and 5 cases (minority class cases), we applied our proposed hierarchical meta-classification approach to separate the records associated with different CKD severity levels.

For each of the methods mentioned above, the training set consisted of records gathered during the first eight years (*2007*–*2014*) while the testing was performed on data gathered during the ninth year (*2015*). As we want to assess the predictive ability of the classifier to infer the evolving stage from temporally earlier data, we do not employ cross-validation for training and testing; rather we train on early data records (collected during the first *8 *years), and test on later records (gathered during the 9^th^ year). To ensure stability of the results, we partitioned the training set stemming from the over-represented class into smaller subsets, by employing multiple random splits, which were used for training the base-classifiers used in the coarse classification stage within the hierarchical meta-classifier (Fig. 1a). The test set was fixed in all experiments to contain all the records collected during the year *2015*.

Performance was evaluated after each classification step using standard measures, namely, *sensitivity, specificity*, *precision* and *F-measure* (see *Testing and Evaluation* sub-section in *Methods*). We compared the performance attained by each of the four base-classifiers (naïve Bayes, logistic regression, decision tree, and random forest) both as baseline and as a component within the simple and the hierarchical meta-classifiers, assessing their respective efficacy in separating CKD stages.

While experiments were performed employing all four base-classifiers, we report here only the results obtained using the random forest, both as a standalone baseline classifier, and when it serves as a component within meta-classification. Using random forest, either alone as a baseline classifier, or as a base-classifier within a meta-classifier, outperforms the other base-classifiers logistic regression, naïve Bayes or decision tree. Similarly, to compare our methods to earlier approaches for addressing imbalance we use random forest classifier in combination with two such earlier approaches, namely, random under-sampling and over-sampling using SMOTE.

**Fig. 3:**
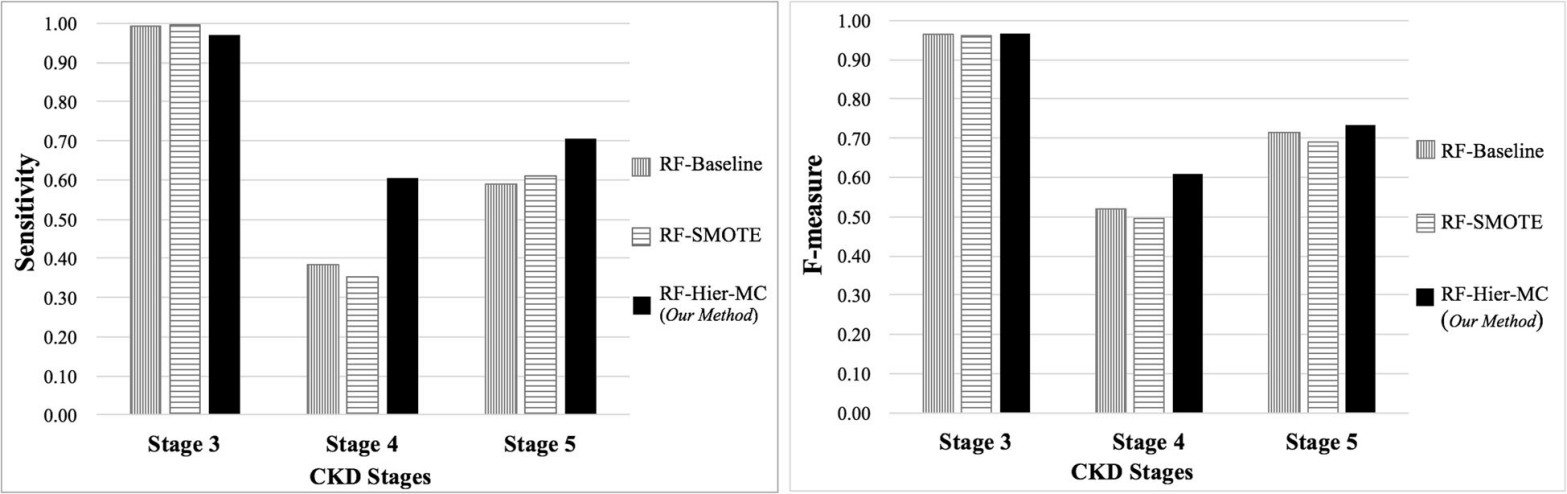
Plots depicting the performance per-class, measured in terms of Sensitivity (left) and F-measure (right), of the random forests baseline classifier (*RF-Baseline,* shown in gray), and of over-sampling using SMOTE (*RF-SMOTE* shown in horizontal stripes) compared with the random forests based *hierarchical meta-classifier* we have developed (*RF-Hier-MC*, shown in black). The X-axes in both plots denote CKD stages; the Y-axes indicate the *sensitivity* (left) and the *F-measure* (right) per stage

Table 4 shows the average specificity, sensitivity and F-measure, attained by our hierarchical meta-classification scheme, compared to those attained by the baseline classifier, the simple, non-hierarchical meta-classifier, the random under-sampling scheme and the over-sampling using SMOTE. Figure 3 shows the sensitivity and F-measure per-class, attained by the baseline classifier, by the over-sampling with SMOTE scheme and by our method. Notably, the figure shows the improved performance of our method for identifying CKD stages *4* and *5*.

**Table 4:** Average specificity, sensitivity and F-measure attained by applying different classification methods to the task of CKD severity level assignment to patients’ records. Results are shown for classifiers developed based on random-forests (*RF*): Our hierarchical meta-classifier (*Hier-MC*), simple meta-classifier – without employing hierarchical stage partitioning (*MC*), Random under-sampling (*Under-Sampling*), Over-sampling using SMOTE (*SMOTE*) and a simple random forests baseline classifier (*Baseline*). Classifiers were trained on office visit records gathered during the period *2007*–*2014,* while records from *2015* were used as the test set. All patient records were represented using the set of *455* features. The highest value for each measure is shown in boldface. Std. deviation is shown in parentheses. See Fig. 3 for detailed analysis of performance per stage

Methods	Sensitivity	Specificity	F-measure
RF-Hier-MC (*Our Method*)	**.93** (0.02)	**.97** (0.02)	**.93** (0.02)
RF-MC	.90 (0.04)	.85 (0.04)	.78 (0.04)
RF-Under-Sampling	.83 (0.08)	.91 (0.07)	.83 (0.08)
RF-SMOTE	.92 (0.06)	.95 (0.06)	.92 (0.06)
RF-Baseline	.92 (0.02)	.94 (0.02)	.92 (0.02)

We also assessed the performance of our classification method while gradually reducing the number of years (and of visit records) included in the patient history. This was done by repeatedly performing each of our experiments, while progressively truncating the early years included in the patient history used as training data, one year at a time, yielding *8* distinct training sets. As described earlier, the test set was kept fixed to include only records collected in *2015*. The first of the training sets included *83,642* records gathered throughout the years *2007–2014*, while the eighth included *19,664* records collected during *2014* alone. The hierarchical meta-classifier was trained using in turn each of the training sets represented by the complete set of *455* features. Performing these experiments helps validate the ability of our classifier to assign the correct severity level even when considering only the most recent history of the patient, which in turn shows generalizability and robustness.

**Fig. 4:**
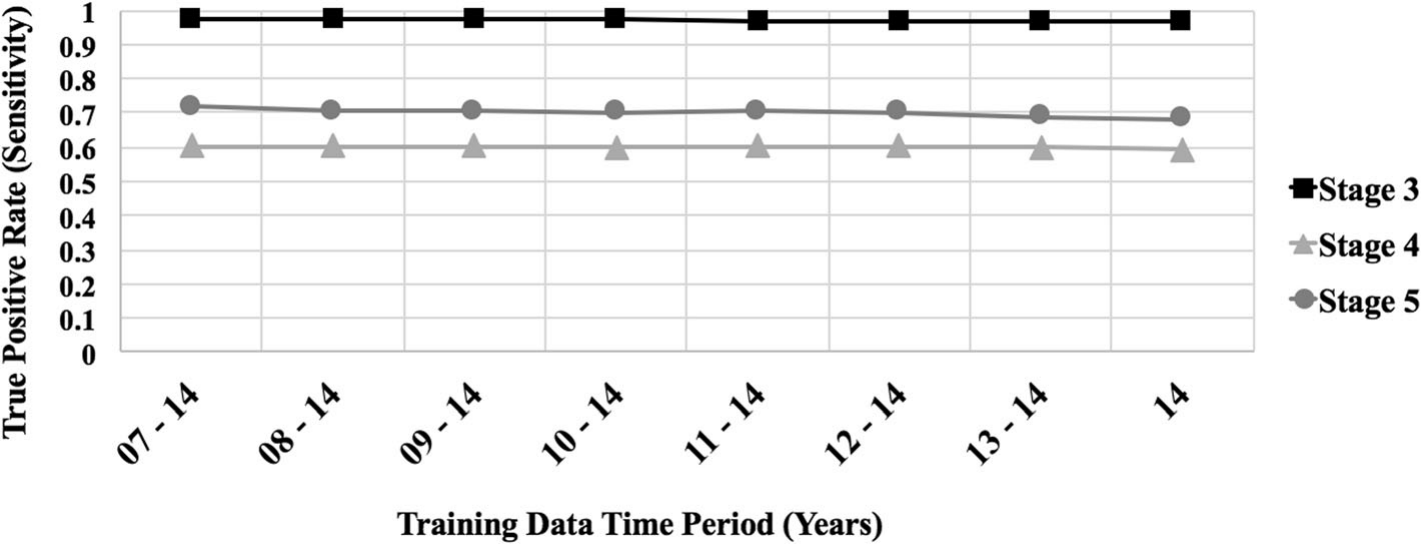
True Positive Rates (TPR), with respect to CKD stages *3*, *4* and *5*, attained by the hierarchical meta-classifiers when trained on datasets obtained by gradually pruning the early years of patient history included in the training set, one year at a time. The X-axis indicates the years covered by each training set, while the Y-axis shows the *true positive rate* (also known as *sensitivity*)

Figure 4 plots the *True Positive Rate* (see Testing and Evaluation sub-section) per-class as a function of the *8* training sets (see Table 3), obtained by progressively pruning, one year at a time, the early years of patient history in the training data. Each of the *eight* sets was used in turn to train the random forest hierarchical meta-classifier. The average *accuracy*, *specificity*, *sensitivity*, *precision* and *F-measure* are all about *0.93* (*std < 0.03*) for all eight sets. As mentioned above, we repeated each experiment *20* times, while employing each time a different split to partition and sub-sample the set of records associated with the over-represented class. The results obtained were similar in all runs, with a small standard deviation (*< 0.03*), thus verifying the stability of the results and of our classification process.

## Discussion

The sampling based ensemble classifier we have introduced attains higher sensitivity with respect to stages *4* and *5* compared to that obtained by other methods (Fig. 3), where other methods fail to identify a large proportion of the CKD stage *4* records and many the stage 5 records. Our proposed method (denoted *RF-Hier-MC*) thus demonstrates improved identification of records associated with severe stages, within the realistic context of highly imbalanced data. Our method also outperforms all other classifiers according to F-measure and specificity (Table 4). We note that the average performance reported in the table of the baseline random forest classifier (denoted *RF-Baseline*) and that of over-sampling with SMOTE based on random forest classifier (denoted *RF-SMOTE*) are similar to that of our method. However, as can be seen in Fig. 3, the performance of the three models varies significantly across the different CKD stages. Our hierarchical meta-classifier clearly shows a higher sensitivity and F-measure for both stage *4* and stage *5* than the simple baseline and the SMOTE classifiers, indicating that our method is more effective than others when identifying each of the two *advanced* CKD stages (stage *4* vs stage *5*). Notably, unlike under-sampling based approaches, our method does not ignore any record associated with the majority class, nor does it create any synthetic sample, as is commonly done in approaches that use over-sampling.

When conducting risk stratification, particular attention must be paid to the avoiding *false negatives* (i.e. the missing a severe case). Not identifying a patient that is in stages *4* or *5* and thus withholding timely proper care carries dire consequences, which are much more severe than those of *false positives* (assigning a stage *4* or *5* label to a stage *3* case). That said, it is still clearly undesirable to cause false alarms by much over-assignment of the more severe labels. To highlight the performance of our method with respect to these issues, we have calculated the *precision*, (also referred to as *positive predictive value*, *PPV*) and *sensitivity* for records associated with stages *4* and 5. *Precision* penalizes for false positives, while *sensitivity* penalizes for false negatives.

Figure 3 clearly demonstrates that compared to other methods, our hierarchical meta-classifier shows a higher sensitivity for records of stage *4* and *5*, while retaining about the same sensitivity level with respect to stage *3*. As for *Precision* with respect to the combined set is *0.76*. That is, of the *1,085* test records classified as stages *4* or *5* by our classifier, *829* are correctly identified. Notably, of the remaining *256* false-positive records, *181* (*~ 70%*) are *borderline cases,* (eGFR values of 30–44, a range typically associated with advanced stage *3* CKD – stage *3b*) [22]. Recent studies indicate that stage *3b* is the inflection point for adverse outcomes, including progression to end stage renal disease (stage *5*) [23]. This observation demonstrates that classifier can identify not only the advanced stage records that have already been identified in our dataset, but also the cases that are likely-to-be severe but were not yet labeled as such.

As indicated by Fig. 4, our classifier’s high performance when trained on datasets obtained by progressively truncating the early years of patient history, one year at time, demonstrate that this model remains effective in distinguishing among the CKD stages even when trained on limited, recent patient history. The figure shows that the true-positive rate remains almost constant, irrespective of the number of years covered by the record; the only exception is a slight decline with respect to stages *4* and *5* for data from *2013*/*14* or *2014* alone. Our classifier’s performance is thus robust, as demonstrated by these results, to reduction in the amount of available patient history.

To summarize, our method demonstrates consistently good performance even when applied to datasets of varying sizes containing office visit records gathered over a limited range of years. As such, our model is stable, generalizable, and likely to be applicable in actual clinical settings, where early records for training are not readily available, while decisions need to be reached based on a relatively brief patient history.

## Conclusion

In this study, we have shown that CKD can be effectively stratified into severity levels using a supervised machine learning method that is based on simple quantitative non-text attributes collected during standard office visits, in the realistic context of highly imbalanced case population. We proposed and developed a sampling based ensemble classification approach, hierarchical meta-classification, to identify CKD stages from a highly imbalanced dataset, achieving high sensitivity, specificity and F-measure, all at or above *0.93*. As demonstrated by our results, our method outperforms baseline classifiers, simple meta-classifier and previously reported approaches for addressing imbalance, in identifying each of the two advanced CKD stages (stage *4* and stage *5*). Moreover, the method maintains its high level of performance when the number of records is significantly truncated, demonstrating its stability and generalizability.

While our proposed sampling-based ensemble method has shown good performance even in the face of data imbalance, the dataset used comprises only records based on data gathered from kidney patients. Future work includes testing and extending the generalizability of our model, using additional datasets in the context of other diseases. We also plan to conduct prospective testing of the model over CKD patients in future studies.

## Abbreviations

CKD: Chronic Kidney Disease
eGFR: Estimated Glomerular Filtration Rate
EHR: Electronic Health Record
KEEP: Kidney Early Evaluation Program
OAA: One-against-all
OAO: One-against-one
PPV: Positive Predictive Value
RF: Random Forest
RF-Hier-MC: Random Forest Hierarchical Meta-Classification
SMOTE: Synthetic Minority Over-Sampling Technique
std.: Standard Deviation
TPR: True Positive Rates
USRDS: United States Renal Disease System

## Footnotes

1 Stage *1* is defined by kidney damage (protein or blood in the urines) while eGFR is normal (eGFR ≥*90 ml/min/1.73m*^*2*^); stage *2* by kidney damage and mildly decreased eGFR (eGFR *60* – < *90*); stage *3* as eGFR *30* – < *60*; stage *4* as eGFR *15* – < *30* and stage *5* as eGFR < *15*.

2 Dataset approved by Christiana Care’s IRB, with a waiver of consent according to 45CFR46.116d.
